# Motor vehicle accidents in CPAP-compliant obstructive sleep apnea patients—a long-term observational study

**DOI:** 10.1007/s11325-020-02023-2

**Published:** 2020-02-14

**Authors:** Minna Myllylä, Ulla Anttalainen, Tarja Saaresranta, Tarja Laitinen

**Affiliations:** 1 Department of Pulmonary Diseases, Division of Medicine, Turku University Hospital and University of Turku, Hämeentie 11, FI-20520 Turku, Finland; 2 Sleep Research Centre, Department of Pulmonary Diseases and Clinical Allergology, University of Turku, Turku, Finland; 3 Hospital Administration, Tampere University Hospital, Tampere, Finland

**Keywords:** Obstructive sleep apnea, Continuous positive airway pressure, Motor vehicle accident, Real-world study

## Abstract

**Purpose:**

Obstructive sleep apnea (OSA) has been associated with a 2- to 7-fold risk of motor vehicle accidents (MVAs). Continuous positive airway pressure (CPAP) treatment may reduce MVA risk. We further explored this issue in long-term CPAP users and untreated controls.

**Methods:**

We used both before-after and case-control study designs. The observational cohort consisted of CPAP-treated and untreated patients matched for gender, age, and apnea-hypopnea index. All MVAs reported to the police were identified.

**Results:**

A total of 2060 patients (75.8% male, mean age 56.0 ± 10.5 years) were included. The CPAP-treated patients (*N* = 1030) were screened for MVAs for a median of 9.0 years before and after treatment. The median CPAP usage was 6.4 h/day. The control patients (*N* = 1030) were screened for MVAs for a median of 6.5 years after discontinuation of CPAP. No significant differences were observed between the incidences of MVAs per 1000 person years before treatment (3.2), after treatment (3.9), or in controls (2.6). Compared with controls, patients who had MVA after treatment had a higher body mass index (BMI), but did not differ in terms of other baseline characteristics, sleep study data, or accident conditions. In the majority of these patients, daytime sleepiness was reduced, whereas BMI tended to increase during treatment.

**Conclusions:**

The MVA incidence did not change after CPAP treatment. Among the patients who had MVA, BMI was the only baseline characteristic that differed between the groups and tended to further increase after CPAP treatment. Differences in sleep study data or accident conditions were not observed.

## Introduction

Obstructive sleep apnea (OSA) has been associated with a 2–7-fold risk of motor vehicle accidents (MVAs) [1]. Body mass index (BMI), excessive daytime sleepiness, apnea-hypopnea index (AHI), oxygen saturation, and changes in brain morphology and neural activation resulting in cognitive impairment may contribute to this risk [1, 2].

In a meta-analysis of nine observational studies (*N* = 1976 in total), MVA risk decreased by 65–78% after CPAP in observation periods of 6 months to 5 years before and after treatment [3]. In one included study, MVAs were reduced both after CPAP and in healthy controls. None of the studies provided objective data on both MVAs and CPAP adherence [3]. Objective data on MVAs has been shown to be more reliable, since OSA patients subjectively reported only one-third of their MVAs, implying their reluctance to disclose their MVA history [4]. In a recent observational study, CPAP use of ≥ 4 h/day was associated with a significantly reduced and lesser use with increased risk of objectively reported MVAs compared with before CPAP [5]. Conversely, a recent randomized controlled trial study found no differences in MVAs among patients treated with CPAP or best supportive care [6]. In both of these studies, less than half of the CPAP-treated patients had used CPAP for ≥ 4 h/day [5, 6].

To overcome limitations of previous studies including inadequate CPAP compliance and lack of long-term data on objectively reported MVAs, we evaluated the incidence of police-reported MVAs in a large cohort of highly adherent OSA patients using both (1) before-after and (2) matched case-control study designs.

## Methods

**Fig. 1:**
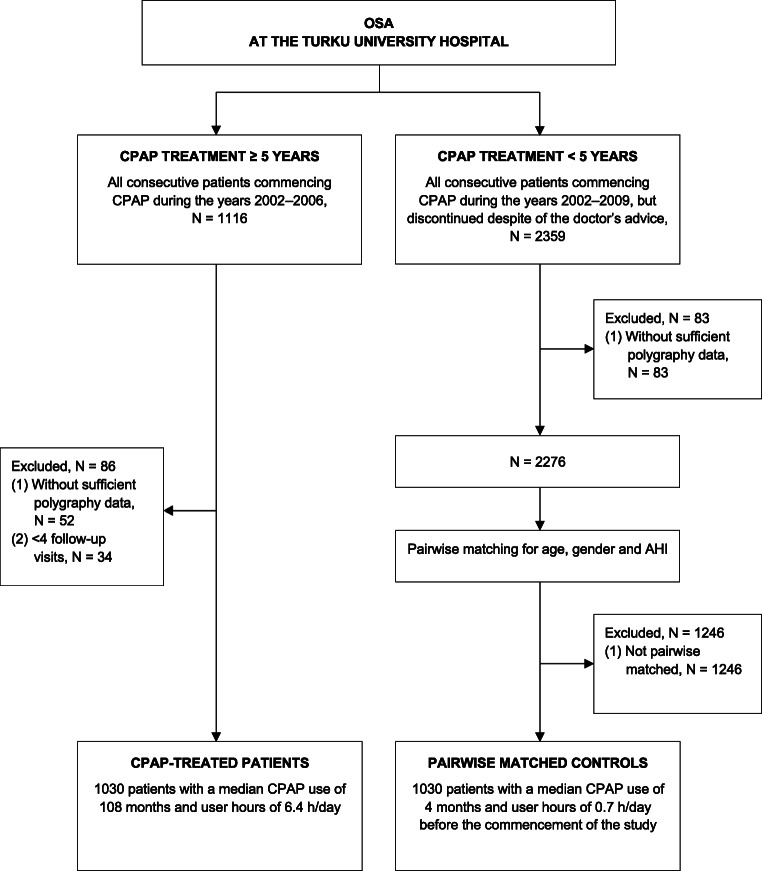
Flowchart of the recruitment of the continuous positive airway pressure (CPAP)-treated obstructive sleep apnea (OSA) patients and their controls matched for age, gender, and apnea-hypopnea index (AHI). Modified and reprinted by permission from Reference [7]

Our age-, gender-, and AHI-matched case-control OSA patient populations have been previously described [7]. All the patients had been treated at the Department of Pulmonary Diseases, Turku University Hospital during 2002–2009 (Fig. 1). Patients without available cardiorespiratory polygraphy data prior to CPAP were excluded. Cases (*N* = 1030) had used CPAP at least for 5 years with at least 4 follow-up visits. All patients in the control group (*N* = 1030) had used CPAP, but despite the doctor’s advice discontinued the treatment within 5 years (median 4 months).

Baseline variables were derived from the electronic medical records [7]. All MVAs reported to the police were identified from the national registry maintained by Statistics Finland. Only accidents involving the study subject as the actual driver were included. In CPAP-treated patients, the median duration of CPAP treatment was 9.0 (IQR 2.5) years with an average of 8.4 ± 2.2 follow-up visits per patient calculated from the commencement of treatment to withdrawal or to the last follow-up visit before the end of 2014. In order to compare MVAs before and after treatment, CPAP-treated patients were also screened for MVAs for 9 years prior to CPAP. The control patients were screened for MVAs for 6.5 years prior to CPAP and for a median of 6.5 (IQR 3.9) years after discontinuation of CPAP calculated from the last CPAP follow-up visit until death or the end of 2014. Data on all-cause death was derived from the national registry. CPAP usage hours (h/day) were recorded by inbuilt counter clock of the CPAP device. For each patient, the mean usage hours across the treatment period were determined.

### Statistical analyses

Data analyses were performed using the IBM SPSS Statistics 25.0 and SAS 9.4 software packages. Normally distributed continuous variables were presented as mean values and standard deviations (SD), and not normally distributed as median values and interquartile ranges (IQR). Means and medians were compared by the independent-sample *T* test and the Mann-Whitney *U* test, respectively, and categorical variables by the χ^2^ test. Cases and controls were matched by using the K-means clustering algorithm with Manhattan distance (absolute value distance). The Poisson regression, in which the individual length of observation was taken into account for each patient, was used for the comparison of MVA incidences between the study groups. *p* values of < 0.05 were considered significant.

## Results

At baseline, CPAP-treated and control patients did not differ in terms of age, gender, or AHI. However, differences in comorbidities, BMI, and smoking habits were observed (Table 1) [7]. In the CPAP-treated patients, the median CPAP use across the 9-year treatment period was 6.4 (IQR 2.3) h/day, and 913 (88.6%) of the patients were compliant to CPAP (≥ 4 h/day). Of the CPAP-treated and control patients, 1.2% and 0.9%, respectively, had engaged in bariatric surgery (*p* = 0.5), and 6.3% of the controls had used a mandibular advancement device (MAD) treatment after CPAP. Data on MAD compliance was not available.

**Table 1:** Baseline characteristics of the studied OSA patients, and comparison of variables between the CPAP-treated patients and controls. Modified and reprinted by permission from Reference 7

	CPAP-treated patients (*N* = 1030)	Control patients (*N* = 1030)	*p* value	All patients (*N* = 2060)
Patients’ characteristics				
Male gender, %	75.8	75.8	1.0	75.8
Age, years (mean, SD)	55.6 ± 9.8	56.4 ± 11.1	0.1	56.0 ± 10.5
BMI, kg/m^2^ (median, IQR)	32.7 (8.1)	31.5 (7.9)	*< 0.001*	32.0 (8.1)
OSA and questionnaires				
AHI, /h (median, IQR)	28.0 (33.0)	27.0 (28.0)	0.1	27.0 (30.0)
ESS score (mean, SD)	9.4 ± 4.7	8.3 ± 4.7	*< 0.001*	8.8 (4.7)
GHQ-12 score (median, IQR)	2.0 (5.0)	2.0 (6.0)	0.1	2.0 (6.0)
Comorbidity and lifestyle				
IFG/T2D, %	40.4	35.2	*0.02*	37.8
Hypertension*, %	76.5	70.3	*0.001*	73.4
Cardiovascular disease^†^, %	5.4	12.6	*< 0.001*	9.0
Psychiatric disorder^‡^, %	15.7	18.3	0.1	17.0
COPD	4.6	7.6	*0.004*	6.1
Smoking, %				
Current smoker	22.0	28.7	*< 0.001*^§^	25.4
Ex-smoker	35.7	34.5		35.1

Data are presented as % or mean ± standard deviation (SD) or median and interquartile range (IQR)

Significant values are shown in italics

*OSA* obstructive sleep apnea, *CPAP* continuous positive airway pressure, *BMI* body mass index, *AHI* apnea-hypopnea index, *ESS* Epworth sleepiness scale (data available for 981 CPAP-treated patients and 986 controls), *GHQ-12* General Health Questionnaire (data available for 912 CPAP-treated patients and 947 controls), *IFG* impaired fasting glucose, *T2D* type 2 diabetes, *COPD* chronic obstructive pulmonary disease

*Blood pressure greater than 140/90 mmHg and/ or use of antihypertensive medication

†Doctor-diagnosed coronary, cerebral, or peripheral artery disease

‡Depression, anxiety, or psychotic disorder

§*p* value for the trend

**Fig. 2:**
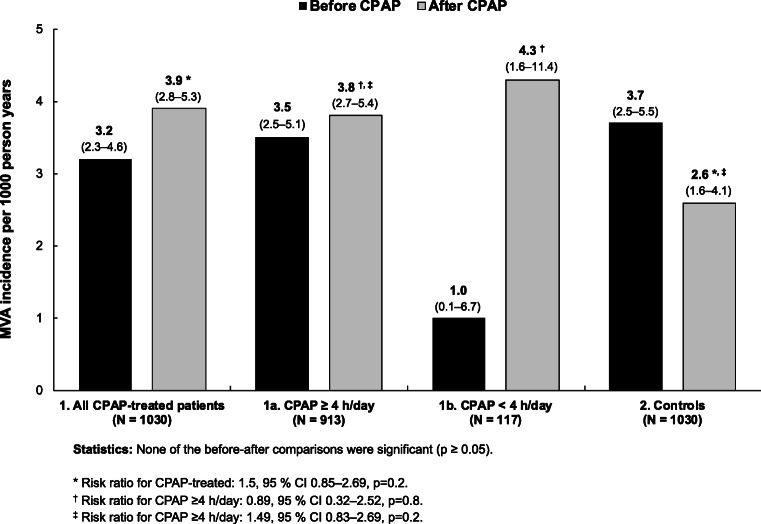
Incidence of motor vehicle accidents (MVAs) per 1000 person years (A) in continuous positive airway pressure (CPAP)-treated patients 9 years before and after the commencement of CPAP treatment in all patients (1), in subgroups of patients with CPAP use of ≥ 4 h/day (1a) or of < 4 h/day (1b), and (B) in control patients 6.5 years before CPAP and after discontinuation of CPAP (2)

Of the 1030 CPAP-treated patients, MVAs were registered in total of 30 patients before and 39 patients after CPAP. Of the latter, 3 MVAs occurred after a several months’ break from CPAP and were thus excluded from the analyses. Of the 1030 controls, MVAs were registered in 17 patients. The incidence of MVAs per 1000 person years was 3.2 (95% CI 2.3–4.6) before, 3.9 (95% CI 2.8–5.3) after CPAP, and 2.6 (95% CI 1.6–4.1) in controls (Fig. 2). Risk for having a MVA did not differ significantly between after and before treatment groups (risk ratio (RR) 1.19, 95% CI 0.73–1.94, *p* = 0.5), or between CPAP-treated and control patients (RR 1.5, 95% CI 0.85–2.69, *p* = 0.2). One patient had two MVAs after CPAP, and one patient had a MVA before and after CPAP. None of the controls had multiple MVAs. Excluding those controls who had used MAD after CPAP did not change the results. Furthermore, the MVA incidence did not differ between the compliant and noncompliant CPAP-treated patients. Among the latter, the incidence tended to increase after CPAP, but the difference was not significant (Fig. 2).

Among the patients with MVA, the only baseline difference was higher BMI in CPAP-treated than in control patients (median 34.2 vs. 31.3 kg/m^2^, *p* = 0.03) (Table 2). The prevalence of cardiovascular disease and the number of professional drivers were low and did not differ between the groups. No differences were observed in sleep study data (Table 2) or accident conditions including the hour of the accident (Table 3). Time to the first MVA, however, was longer among CPAP-treated than that of among controls (median 42.5 vs. 15.0 months, *p* = 0.02). In the CPAP-treated patients with MVA, a mean increase of 1.2 ± 3.7 kg/m^2^ units and a mean decrease of 3.7 ± 3.6 scores were observed during treatment in BMI and Epworth sleepiness scale (ESS) score, respectively. The median use of CPAP did not differ between CPAP-treated patients with or without MVA (median 6.4, IQR 2.2 vs. 6.4, IQR 2.3 h/day, *p* = 0.8).

**Table 2:** Comparison of the characteristics between the CPAP-treated and control patients with motor vehicle accident, and between the patients with and without motor vehicle accident

	CPAP vs. controls			MVA vs. without MVA		
	MVA CPAP (*N* = 36)	MVA controls (*N* = 17)	*p* value	MVA total (*N* = 53)	Without MVA (*N* = 2007)	*p* value
Patients’ characteristics						
Male gender, %	91.7	100.0	0.2	94.3	75.3	*< 0.001*
Age, years (mean, SD)	51.5 ± 10.1	49.6 ± 12.3	0.6	50.9 ± 10.8	56.1 ± 10.4	*< 0.001*
BMI, kg/m^2^ (median, IQR)	34.2 (6.7)^‡^	31.3 (7.5)	*0.03*	33.2 (7.6)	32.0 (8.1) ^‡^	0.3
OSA and questionnaires						
AHI, /h (median, IQR)	28.0 (28.5)	25.0 (38.0)	0.9	27.0 (28.5)	28.0 (30.0)	0.4
ODI4/5, /h (median, IQR)	14.6 (25.4)	21.1 (26.8)	0.8	15.1 (24.1)		
SpO2, %, (median, IQR)						
Mean	93.5 (2.8)	93.3 (3.1)	0.7	93.4 (3.0)		
Minimum	82.0 (10.3)	78.0 (11.0)	0.4	81.3 (11.0)		
T90	5.5 (15.2)	8.5 (31.4)	0.6	6.2 (24.4)		
ESS score (mean, SD)	8.8 ± 3.9	6.4 ± 4.8	0.06	8.1 ± 4.3	8.8 ± 4.7	0.2
GHQ-12 score (median, IQR)	1.0 (6.0)	1.0 (4.0)	0.4	1.0 (6.0)	2.0 (6.0)	0.2
Comorbidity and lifestyle						
IFG/T2D, %	41.7	29.4	0.4	37.7	37.8	1.0
Hypertension/CVD*, %	61.1	70.6	0.5	64.2	73.6	0.1
Psychiatric disorder^†^, %	22.2	17.6	0.7	20.8	16.9	0.5
Smoking, %	41.7	47.1	0.7	43.4	24.9	*0.002*

Data are presented as % or mean ± standard deviation (SD) or median and interquartile range (IQR)

Significant values are shown in italics

*MVA* motor vehicle accident; *CPAP* continuous positive airway pressure; *BMI* body mass index; *OSA* obstructive sleep apnea; *AHI* apnea-hypopnea index; *ODI4/5* oxygen desaturation index of 4%, or alternatively, of 5% (data available for 34 CPAP-treated and 16 control patients); *SpO2* level of blood oxygen saturation (data on mean and minimum available for 34 of the CPAP-treated and 15 of the control patients); *T90* percentage of time spent under SpO2 of 90% (data available for 26 of the CPAP-treated and 12 of the control patients); *ESS* Epworth sleepiness scale (data available for 36 CPAP-treated patients, 16 controls, and 1915 patients without MVA); *GHQ-12* General Health Questionnaire (data available for 32 CPAP-treated patients, 15 controls, and 1812 patients without MVA); *IFG* impaired fasting glucose; *T2D* type 2 diabetes

*Blood pressure greater than 140/90 mmHg and/ or use of antihypertensive medication or doctor-diagnosed coronary, cerebral or peripheral artery disease

†Depression, anxiety, or psychotic disorder

‡BMI of the CPAP-treated patients with MVA compared with that of patients without MVA, *p* = 0.04

**Table 3:** Comparison of the characteristics of motor vehicle accidents between the CPAP-treated and control patients

	MVA CPAP (*N* = 36)	MVA controls (*N* = 17)	*p* value
Time to the accident, months* (median, IQR)	42.5 (48.5)	15.0 (41.5)	*0.020*
Time of day, hour, %			0.6^§^
8:00 p.m.–7:59 a.m.	22.2	29.4	
8:00 a.m.–7:59 p.m.	77.8	70.6	
Autumn/winter, %	50.0	47.1	0.8
Weather/road surface condition worsen^†^, %	44.4	29.4	0.3
Speed limit ≥ 60 km/h, %	41.7	47.1	0.7
Outside urban area, %	44.4	41.2	0.8
Junction or traffic lights, %	41.7	58.8	0.2
Alcohol/drug/medicine use, %	11.1	17.6	0.5
DL CDE, %	62.9	66.7	0.8
Vehicle type^‡^, %			0.9^§^
Car, moped, or motorcycle	83.3	82.4	
Heavy vehicle	16.7	17.6	
Injured, %	38.9	41.2	0.9

Data are presented as % or median and interquartile range (IQR)

Significant values are shown in italics

*MVA* motor vehicle accident, *CPAP* continuous positive airway pressure, *DL* driver’s license (data missing for 1 CPAP-treated and 5 control patients)

*Time from the commencement of CPAP treatment (CPAP-treated patients) or from the last CPAP follow-up visit (controls) to the occurrence of first motor vehicle accident

†The presence of fog, rain or snow, or road surface bare and wet, snowy or icy

‡Car including passenger car, van, and truck ≤ 3500 kg; heavy vehicle including truck > 3500 kg, bus

§*p* value for the trend

## Discussion

In this large long-term observational study, MVA incidence did not change when compared before and after CPAP, compliant and non-compliant CPAP users, or CPAP-treated and untreated patients. Similarly, in a previous observational study by Català et al., MVAs did not differ between 1 year before and after treatment in patients with CPAP use of ≥ 4 h/day or less [8]. However, the result of the present study differed from most previous studies and was to some extent unexpected. Despite good CPAP compliance, the sleep duration of the CPAP-treated patients may have been insufficient, thus predisposing to MVAs. MVA data was based on a national registry in which small, near miss accidents or close shaves on the road are not reported. In addition, we do not know the actual annual driving exposure in the cohort. In general, 93% of the Finnish population aged 50–54 years has a valid driver’s license [9–11]. CPAP-treated and control patients were matched for age and gender, which likely decreases differences in driving habits. Potential differences should not have an effect on before and after comparisons.

As a further limitation, data on changes in BMI and ESS score was not available for controls since they were followed through the MVA registry. Even though the controls were selected as carefully as possible, there may be some underlying patient characteristics including different phenotypes of OSA and personal types causing bias in the results, since patients compliant to CPAP may differ from those who refuse to continue CPAP. All the patients had been diagnosed and treated at one clinic using the same protocol and treatment guidelines. Similar to previous studies, patients with MVA were more frequently male [12], younger [5, 12], and had a higher prevalence of smokers [13] than those without MVA (Table 2). Smoking has been associated with an increased risk of MVAs possibly due to smoking-related diseases, distractibility, and smokers’ susceptibility to risky behavior [13]. We also showed that accident conditions, a possible confounding factor for MVAs, did not differ between the CPAP-treated patients and controls, which to our knowledge have not been assessed before.

Interestingly, time to the first MVA was almost three times longer among CPAP-treated than those among controls. In further analysis, the MVA incidence did not, however, significantly differ between the groups when the length of observation was reduced in both groups to 2, 3.5, or 5 years. Furthermore, daytime sleepiness tended to decrease in the majority of the patients who had MVA after CPAP. It has been actually suggested that cognitive impairment and daytime sleepiness may not be fully normalized by CPAP despite good treatment compliance [14]. However, current data on the association between long-term CPAP treatment and cognitive changes is scarce, and the possible effect of the residual cognitive impairment on the results of the present study remains as a speculation. Baseline BMI was also higher among the OSA patients who adapted to CPAP than those who did not, and tended to further increase during treatment among the patients with MVA. Adipocytes have shown to secrete cytokines, which may increase daytime sleepiness and thus predispose to MVAs [15].

## Conclusion

Despite good CPAP compliance, the MVA incidence did not differ between before and after CPAP or between CPAP-treated and untreated OSA patients. Apart from BMI, differences in baseline characteristics, sleep study data, or accident conditions were not observed between the CPAP-treated and control patients with MVA. The results remain to be confirmed in further studies with even longer follow-ups, objective data on MVAs, patients compliant to CPAP, and data on both actual annual driving exposure and changes in BMI and cognition.
